# Comparison of transvaginal natural orifice transluminal endoscopic surgery and laparoendoscopic single-site surgery for tubal pregnancy

**DOI:** 10.1097/MD.0000000000045770

**Published:** 2025-11-07

**Authors:** Panpan Bei, Juan Li, Xiaoyan Huang, Yuli Bi, Wei Wang, Hang Yuan, Ting Qiu, Yujie Su, Jidong Wang

**Affiliations:** a Department of Gynaecological Oncology Comprehensive Centre, Shandong Provincial Maternal and Child Health Care Hospital Affiliated to Qingdao University, Jinan, China; b Department of Clinic, Shandong Provincial Maternal and Child Health Care Hospital Affiliated to Qingdao University, Jinan, China.

**Keywords:** LESS, minimally invasive surgery, surgical outcomes, v-NOTES, tubal pregnancy

## Abstract

Transvaginal natural orifice transluminal endoscopic surgery (v-NOTES) offers several unique advantages including reduced postoperative pain and improved cosmetic outcomes. This study aimed to evaluate the effectiveness of v-NOTES in the treatment of tubal pregnancy, with a focus on patient outcomes and cost-effectiveness. A retrospective analysis was conducted on 40 cases of tubal pregnancy treated surgically between December 1, 2020 and May 31, 2022. Patients were divided into 2 groups: the v-NOTES group (n = 20), who underwent transvaginal natural orifice transluminal endoscopic surgery, and the control group (n = 20), who underwent laparoscopic single-site surgery (LESS). Key variables including age, body mass index, operative time, intraoperative blood loss, postoperative pain, recovery time, and total costs were evaluated and compared between the 2 groups. Operative time and intraoperative blood loss were comparable between the 2 groups, patients in the v-NOTES group reported significantly lower postoperative pain scores and shorter hospital stays (*P* < .05). Additionally, the time return to normal activity was markedly shorter in the v-NOTES group compared to the LESS group (16 hours vs 25 hours, respectively). Both procedures demonstrated similar safety profiles; but v-NOTES was associated with a faster recovery and higher patient satisfaction. V-NOTES is a safe and effective minimally invasive surgical option for tubal pregnancy, providing enhanced recovery and improved patient experience compared to LESS.

## 1. Introduction

Minimally invasive surgical techniques have undergone significant advancements in recent years, particularly in the management of gynecological disorders.^[1]^ A major driver of this progress has been the adoption of enhanced recovery after surgery (ERAS) protocols, which focus on optimizing surgical outcomes and expediting postoperative recovery through a comprehensive multidisciplinary approach.^[2,3]^ These evidence-based protocols encourage the refinement of surgical techniques to not only minimize invasiveness, but also maximize patient safety, reduce recovery times, and enhance overall surgical efficacy. Minimally invasive laparoscopic techniques have gained widespread acceptance in the surgical treatment of gynecological conditions.^[4,5]^ Their popularity stems from well-established benefits, including smaller incisions, reduced postoperative pain, shorter hospital stays, and faster recovery.^[6]^ These advantages align closely with the goals of ERAS, and have made laparoscopy the gold standard for the management of several gynecological procedures. Additionally, laparoscopic surgery offers “optimal stability and minimal scarring,” improving not only clinical outcomes but also cosmetic satisfaction, which is an increasingly important consideration for patients.^[7,8]^

Building upon the success of laparoscopy, more advanced techniques have emerged, such as laparoendoscopic single-site surgery (LESS) and natural orifice transluminal endoscopic surgery (NOTES). These next-generation approaches aim to further reduce abdominal wall trauma by minimizing or eliminating visible incisions, thereby improving both recovery and patient experience.^[9–11]^ LESS involves performing surgery through a single umbilical incision, enhancing cosmetic outcomes and reducing invasiveness.^[12,13]^ However, even this single-site abdominal access can still be associated with risks, including infection and trocar-site herniation.^[14]^

In response to these limitations, NOTES was developed using natural orifices such as the mouth, anus, vagina, and urethra to access internal surgical sites without the need for any external incisions.^[15]^ This novel strategy minimizes the risks associated with abdominal wall entry. Among the various types of NOTES, transvaginal NOTES (v-NOTES) has garnered particular attention in gynecological surgery.^[16]^ By accessing the pelvic cavity through the vaginal route, v-NOTES eliminates the need for abdominal trocars, offering distinct advantages over traditional laparoscopy.^[17,18]^ Notably, v-NOTES avoids visible scars and is associated with less postoperative pain, faster recovery, and shorter hospital stays compared to conventional laparoscopy.^[19–21]^ These outcomes are closely aligned with ERAS principles and reflect the patient-centered philosophy that increasingly guides modern surgical practice.

Despite these advantages, v-NOTES remains considerably less utilized in routine clinical practice than traditional laparoscopy, largely due to its relative novelty and technical challenge. This study was designed to compare and analyze the application of v-NOTES and LESS in the surgical treatment of tubal pregnancy, with a focus on surgical indications, outcomes, complications, and techniques.

## 2. Materials and methods

### 2.1. Ethical considerations

This study was approved by the Ethical Review Board of Shandong Province Maternal and Child Health Hospital. All patient data were anonymized, and financial records were accessed with permission from the relevant hospital departments.

### 2.2. Study design

This single-center, retrospective cohort study was conducted at Shandong Province Maternal and Child Health Care Hospital between December 1, 2020 and May 31, 2022. A total of 40 women diagnosed with tubal ectopic pregnancy underwent either v-NOTES (n = 20) or LESS (n = 20). All surgeries were performed by the same experienced gynecologist to ensure consistency in techniques and outcomes. Data collected included patient demographics, perioperative details, postoperative recovery parameters, and overall treatment costs.

### 2.3. Patient selection

The inclusion criteria were confirmed tubal pregnancy and suitability for either v-NOTES or LESS. The exclusion criteria included uterine prolapse or severe pelvic adhesions, while a history of pelvic surgery was not considered a contraindication. Patients were evaluated preoperatively through pelvic examination, transvaginal ultrasound, and dynamic blood human chorionic gonadotropin monitoring.

### 2.4. Surgery performance

**Figure 1.**
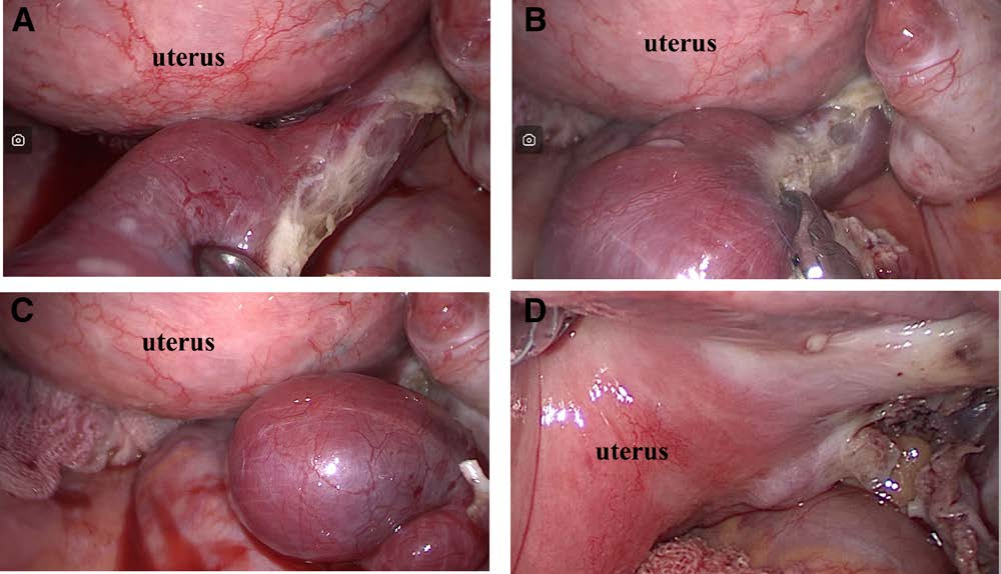
Salpingectomy via v-NOTES. (A) Expose the fallopian tube on the affected side for clear visualization. (B) Make an incision along the mesosalpinx extending to the uterine horn. (C) The fallopian tube was completely removed, revealing an enlarged, purple–blue ampulla containing ectopic pregnancy tissue. (D) Pelvic findings following resection of the fallopian tube. v-NOTES = transvaginal natural orifice transluminal endoscopic surgery.

V-NOTES salpingectomy: Patients were placed in the lithotomy position under general anesthesia. After routine disinfection, gynecological examination was performed to determine the position of the uterus. The posterior lip of the cervix was clamped using Allis forceps, and a 1.0 cm incision was made in the posterior vaginal fornix, approximately 2.0 cm in length. A transvaginal laparoscopic access device was then inserted and insufflation was initiated at 8 mm Hg. The affected fallopian tube was excised along the mesosalpinx after exploration of the peritoneal cavity. Finally, 2–0 absorbable sutures (Ethicon, USA) were used to close the incision (Fig. 1).

**Figure 2.**
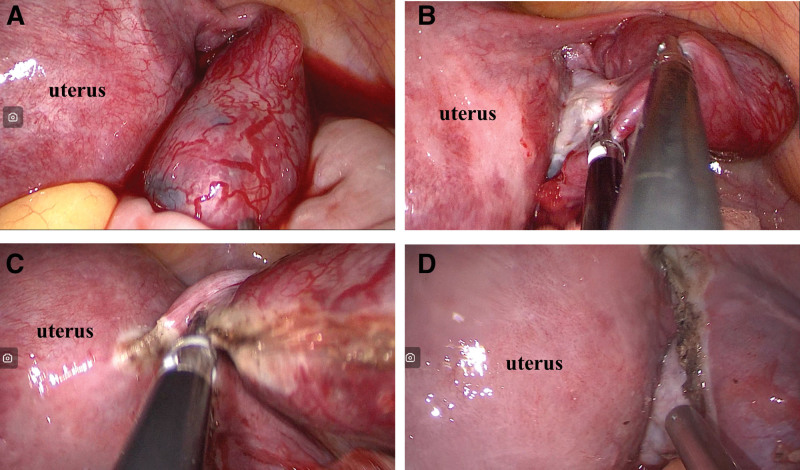
Salpingectomy via LESS. (A) Right fallopian tube ampullary pregnancy. (B) Separation of adhesions between the fallopian tube and ovary. (C) Make an incision along the mesosalpinx extending to the uterine horn. (D) Outcome following fallopian tube resection. LESS = laparoscopic single-site surgery.

LESS salpingectomy: All operations were performed under general anesthesia with the patient in the supine position. A laparoscopic access device was inserted through the umbilicus into the pelvic cavity, and insufflation was initiated at 12 to 15 mm Hg. The surgical procedure was similar to that of v-NOTES, and all surgeries were performed by the same experienced gynecologist (Fig. 2).

### 2.5. Clinical outcome measures

The primary outcomes included operative time, blood loss, postoperative pain (measured using the visual analog scale at 6, 12, and 24 hours postoperatively), postoperative infection, hospital stay, and return to normal activities. Costs were also analyzed to assess the cost-effectiveness.

### 2.6. Patient-reported outcomes

In addition to clinical metrics, patient-related outcomes were collected to evaluate subjective recovery experience, pain perception, and satisfaction with the surgical procedure. The questionnaires included the following measures: Visual analog scale for pain assessment at 6, 12, and 24 hours postoperatively; Return to normal activities; Patient satisfaction questionnaire: A survey to gauge overall satisfaction with the surgical procedure, addressing factors such as pain management, recovery experience, and cosmetic results at 1and 3 months postoperatively.

These patient-related outcomes were analyzed to compare the patient experiences between the v-NOTES and LESS groups.

### 2.7. Statistical analysis

IBM SPSS Statistics for Windows, Version 20.0 (IBM Corp., Armonk) was used for the data analysis. Continuous variables were assessed for normality using the Shapiro–Wilk test. Nonnormally distributed data are summarized using the median (range). Categorical variables are presented as frequencies and percentages. The Mann–Whitney *U* test was used to compare continuous variables. The chi-square test was used to compare the proportions of categorical variables. Statistical significance was set at *P* < .05.

## 3. Results

### 3.1. Baseline characteristics

There were no significant differences in body mass index between the groups (*P* > .05). The v-NOTES group had significantly older patients (35.5 years vs 32 years, *P* = .017) and higher parity (1.5 vs 1, *P* = .000) than the LESS group. In the v-NOTES group, all patients who had no plans for future pregnancies underwent salpingectomy, whereas more than half of the patients in the LESS group were required to preserve the affected fallopian tube because of the desire to have more children (55%). Patients in the LESS group were generally younger and had fertility plans, reflecting the impact of the choice of surgical method on the study outcomes(table 1).

**Table 1:** Patient demographics and preoperative characteristics.

Characteristics	v-NOTES salpingectomy	LESS salpingectomy	*P* value
Sample size (n)	20	20	
Age (yr)	35.5 (33, 38.75)	32.0 (29.25, 35)	.017
BMI (kg/m^2^)	23.24 (20.80, 24.40)	24.22 (21.52, 25.57)	.561
Surgical method			
Salpingectomies	20 (100%)	9 (45%)	
Tube-sparing	0 (0%)	11 (55%)	
Parity	1.5 (1, 2)	1.0 (0, 1)	.000
Living offspring			
0	1 (5%)	9 (45%)	
1	9 (45%)	10 (50%)	
2	10 (50%)	1 (5%)	

Values are expressed as median (range) or numbers (percentage).

BMI = body mass index, LESS = laparoscopic single-site surgery, v-NOTES = transvaginal natural orifice transluminal endoscopic surgery.

### 3.2. Surgical outcomes

There were no significant differences in the operative time or intraoperative blood loss between the v-NOTES and LESS (table 2). However, the postoperative pain scores were significantly lower in the v-NOTES group (*P* = .000, Table 2). v-NOTES patients returned to normal activities faster, within 16 hours, compared to 25 hours in the LESS group (*P* = .000). The v-NOTES group also had shorter hospital stays (2 vs 4 days, *P* = .000), emphasizing the advantages of this technique in accelerating postoperative recovery (table 2).

**Table 2:** Intraoperative and postoperative outcomes.

Surgical outcomes	v-NOTES salpingectomy	LESS salpingectomy	*P* value
Operative time (min)	78.5 (60, 110)	70 (60, 75.75)	.146
Blood loss (mL)	20 (10, 45)	20 (10, 20)	.638
Postoperative pain (VAS scores)			
6 h	1 (1, 2)	3.5 (3, 4)	.000
12 h	1 (0, 1)	2 (2, 3)	.000
24 h	0 (0, 0)	1 (1, 2)	.000
Return to normal activity (h)	16 (13, 20)	25 (18, 30)	.000
Postoperative infection (cases)	0 (0, 0)	0 (0, 0)	
Hospital stays (d)	2 (2, 3)	4 (3, 4)	.000
Total costs ($)	2136 ± 159.1	2055 ± 126.6	.083

The total costs are expressed mean ± SD and other values are expressed as median (range).

LESS = laparoscopic single-site surgery, VAS = visual analog scale, v-NOTES = transvaginal natural orifice transluminal endoscopic surgery.

### 3.3. Patient satisfaction and postoperative complications

Patients undergoing v-NOTES reported higher satisfaction with the procedure, particularly in terms of pain management and recovery. No significant differences in complication rates were observed between the 2 groups, further supporting the safety of v-NOTES.

### 3.4. Cost analysis

Although there was no significant difference in the total costs between the 2 groups (*P* = .083), the faster postoperative recovery observed in the v-NOTES group may lead to reduced indirect costs—such as fewer days off work and a quicker return to daily activities. This finding suggests that v-NOTES may provide better value over time (table 2).

## 4. Discussion

Minimally invasive techniques have evolved significantly over the past 2 decades, providing substantial benefits such as smaller incisions, reduced pain and trauma, fewer complications, shorter hospital stay, faster recovery, and improved cosmetic outcomes.^[22]^ Gynecological laparoscopic surgery has made continuous advancements, driven by increasingly sophisticated technologies. However, the ongoing challenge is to further minimize invasiveness while enhancing patient-centered care.^[21,23]^ As laparoscopic techniques have advanced, particularly with the growing use of LESS, a new vaginal approach has emerged: v-NOTES.^[24]^ This innovative method eliminates the need for traditional abdominal incisions, making it even more minimally invasive and cosmetically favorable, while reducing complications associated with trocar use in conventional laparoscopic surgery.^[1,25,26]^

The results of this study demonstrated that v-NOTES offers significant benefits over LESS for the treatment of tubal pregnancy, particularly in terms of postoperative pain, recovery time, and patient satisfaction. Despite comparable surgical times and blood loss, the v-NOTES group experienced a markedly faster recovery, reduced hospital stays, and earlier return to normal activities, aligning with ERAS protocols aimed at minimizing surgical trauma and optimizing recovery. From a cost perspective, although the direct medical costs between the 2 procedures were not significantly different, the shorter hospital stay, and quicker recovery associated with v-NOTES may have led to lower overall healthcare costs. Given faster recovery and higher patient satisfaction, v-NOTES has emerged as a potentially more cost-effective alternative, particularly for patients who prioritize minimal postoperative pain and rapid return to daily life.

The findings of this study align with previous research, suggesting that v-NOTES offers a more patient-centered approach to minimally invasive surgery by eliminating visible scars and reducing postoperative discomfort. However, the learning curve associated with v-NOTES remains a challenge, requiring additional training of surgeons to ensure optimal outcomes.^[27]^ Transvaginal entry into the abdominal cavity is a key challenge in v-NOTES. This approach requires precise anatomical knowledge and advanced surgical skills to avoid injury to surrounding structures during access. The confined workspace, limited range of instrument movement, and reliance on indirect visualization add to the complexity of the procedure. These technical demands make v-NOTES more challenging than traditional laparoscopic methods, necessitating specialized training to achieve safe and effective outcomes.^[28]^

Given the limited operating space in v-NOTES, which increases the risk of intestinal injury, we used a saline-soaked gauze roll during the procedure. This technique not only isolates the intestines effectively from surgical instruments but also provides certain support to the uterus. This not only expands the surgical operating space but also reduces the chance of intestinal damage.

The retrospective nature of the study and its relatively small sample size are its limitations. The study was conducted at a single-center, which may limit the generalizability of the findings.

## 5. Conclusion

V-NOTES for tubal pregnancy represents an innovative technique in the field of minimally invasive gynecologic surgery, offering clear benefits over LESS in terms of patient recovery and satisfaction. Mastery of surgical skills, particularly suturing techniques, is essential to achieve optimal outcomes. Further research with larger sample sizes and multicenter studies are needed to confirm these findings and establish v-NOTES as a standard surgical option for tubal pregnancies.

## Acknowledgments

We would like to express our gratitude to ChatGPT, developed by OpenAI, for assisting with language refinement and for improving the clarity of this manuscript.

## Author contributions

**Data curation:** Panpan Bei, Juan Li.

**Formal analysis:** Yuli Bi.

**Funding acquisition:** Jidong Wang.

**Investigation:** Ting Qiu.

**Project administration:** Panpan Bei, Juan Li, Xiaoyan Huang, Yujie Su, Jidong Wang.

**Supervision:** Jidong Wang.

**Visualization:** Wei Wang.

**Writing – original draft:** Panpan Bei, Hang Yuan.

**Writing – review & editing:** Jidong Wang.
